# Human NKT10 cells are enriched in cord-derived invariant natural killer T cells and mediate immune-regulation in a xenogeneic graft-versus-host disease model

**DOI:** 10.3389/fimmu.2026.1834739

**Published:** 2026-06-10

**Authors:** Abel Trujillo-Ocampo, Pamella Borges, Maison Grefe, Martiela Vaz de Freitas, Sung-Eun Lee, Yuan Qi, Jelita Clinton, Dan Li, Hong He, Ling Yu, Arnau Peris-Cuesta, Erik A. Ehli, Qing Ma, Xiaoping Su, Dinler Amaral Antunes, Gheath Al-Atrash, Jeffrey J. Molldrem, Elizabeth J. Shpall, Jin S. Im

**Affiliations:** 1 Department of Hematopoietic Biology and Malignancy, The University of Texas MD Anderson Cancer Center, Houston, TX, United States; 2 Department of Biology and Biochemistry, The University of Houston, Houston, TX, United States; 3 Department of Hematology, Seoul St. Mary’s Hospital, College of Medicine, The Catholic University of Korea, Seoul, Republic of Korea; 4 Department of Bioinformatics and Computational Biology, The University of Texas MD Anderson Cancer Center, Houston, TX, United States; 5 Department of Therapeutic Discovery, The University of Texas MD Anderson Cancer Center, Houston, TX, United States; 6 Avera McKennan Hospital and University Health Center, Sioux Falls, SD, United States; 7 Department of Stem Cell Transplantation and Cellular Therapy, The University of Texas MD Anderson Cancer Center, Houston, TX, United States

**Keywords:** cord-derived iNKT cells, GvHD, immune-regulation, NKT10, transplantation

## Abstract

CD1d-restricted invariant natural killer (iNK) T cells are innate T cells known for their ability to shape adaptive immunity toward inflammation or immune-suppression via the rapid production of Th1-, Th2-, and Th17-type cytokines from corresponding iNKT subsets such as NKT1, NKT2, and NKT17. IL-10-producing invariant NKT cells, termed NKT10 cells, are thought to play an immunoregulatory role, but their potential clinical use remains underexplored. We characterized human NKT10 cells from cord-derived iNKT cells and investigated their therapeutic utility in allogeneic stem cell transplantation. Cord and cord-derived iNKT cells contained a high frequency of CD4^+^CD25^+^CD161^low^FoxP3^+^ iNKT cells and showed Th2/Th10-biased cytokine production upon antigenic stimulation. Accordingly, cord-derived iNKT cells displayed a distinct gene expression profile with upregulated genes related to NKT2, NKT10, and regulatory T cells compared with adult donor-derived iNKT cells. Furthermore, single-cell RNA sequencing analysis of cord-derived iNKT cells confirmed the presence of NKT10-like subset that was enriched with multiple immunoregulatory pathways and genes related to immune-checkpoints (*NRP1*, *PD1*, *CLTA-4*, and *GITR*) and NKT10 (*MAF*, *HIF1A*, and *FoxP3*), whereas the NKT1/17-like subset present in adult donor-derived iNKT cells showed upregulation of genes related to cytotoxicity (*GZMA/B*, *KLRD1*, and *PRF1*), NKR (*KLRK1*, *KLRB1*, *KLRG1*, and *NKG7*), NKT1 (*EOMES* and *TBX21*), and NKT17 (*RORC*). Lastly, cord-derived iNKT cells suppressed alloreactive T cell proliferation *in vitro* and ameliorated xenogeneic graft-versus-host disease where the immunodeficient NSG mice received human peripheral blood mononuclear cells supplemented with cord-derived iNKT cells. Thus, NKT10-enriched, cord-derived iNKT cells are candidate cell therapeutics for immune-modulation in allogeneic stem cell transplantation and other autoimmune diseases.

## Introduction

Invariant natural killer T (iNKT) cells are a unique subset of T lymphocytes that recognize glycolipid antigens presented on the non-polymorphic major histocompatibility complex class I-like molecule CD1d through the semi-invariant T cell antigen receptor, invariant Vα14Ja18 (TRAV11^+^TRAJ18^+^) paired with Vβ8, Vβ7, and Vβ2 in mice, and Vα24Ja18 (TRAV10^+^TRA18^+^) paired with Vβ11 in humans (1, 2). Upon activation, iNKT cells can influence adaptive immunity toward either immune-suppression or inflammation via the production of Th1- and Th2-type cytokines and enhance adaptive T- and B-cell responses via the CD40–CD40L feedback loop with antigen-presenting dendritic cells (DCs) (3, 4). Furthermore, iNKT cells constitutively express cytolytic granules similar to NK cells and display potent cytolytic activity against target cells in an antigen-dependent manner (5, 6). Thus, iNKT cells may play a critical role in orchestrating adaptive immunity.

The iNKT cells are heterogeneous in phenotype (CD4^+^ vs. CD4^-^) and function [NKT1 (Th1), NKT2 (Th2), NKT10 (Th10), and NKT17 (Th17)] (7–18), and the developmental pathways and roles of the murine iNKT cells have been well documented in innate and adaptive immunity (10, 13–18). The presence of multiple iNKT functional subsets explains the functional plasticity of iNKT cells, which exert a range of immunomodulatory effects. These functional subsets are differently distributed between CD4^+^ and CD4^-^ iNKT cells—for example, NKT2/10-enriched CD4^+^ iNKT cells express CD25 and Foxp3 and produce Th2 cytokines better than CD4^-^ iNKT cells, whereas NKT1/17-enriched CD4^-^ iNKT cells express higher levels of cytotoxic granules (perforin and granzyme), activating NK receptors and chemokine receptors, and produce Th1 cytokines better than CD4^+^ iNKT cells. This intrinsic functional polarization classifies CD4^+^ iNKT cells as better regulators and CD4^-^ iNKT cells as better effectors, although iNKT cells have overlapping functions.

Although the immunostimulatory effector function of iNKT cells and their mechanism of action have been extensively investigated and are foundational for iNKT-targeted therapeutics (3, 19–24), how iNKT cells coordinate immune-suppression is under-studied. Recently, the murine IL-10 -producing iNKT subset, NKT10 cells, has been described in mice pretreated with the agonist glycolipid, alpha-galactosyl ceramide (αGalCer) (15). NKT10 cells are reported to play a regulatory role in the pathogenesis of obesity (16–18), diabetes (17, 18, 25), experimental autoimmune encephalomyelitis (15, 26), tumor progression (15), and colitis (27). Thus, NKT10 cells play a critical role in the regulatory function of CD4^+^ iNKT cells. Unlike the well-defined murine NKT10 cells (15, 28), the phenotype and function of IL-10-producing human iNKT cells are less well-characterized, including their regulatory role in human diseases. Further investigation to define human NKT10 cells is needed to develop NKT10-directed immunotherapeutics to mitigate various inflammatory diseases. Because cord blood reportedly contains a high fraction of the CD4^+^ iNKT subset (29, 30), we sought to delineate human NKT10 cells from cord-derived iNKT cells and evaluate their immunoregulatory function in the context of allogeneic stem cell transplantation (ASCT).

ASCT is a curative therapy for patients with blood cancer or disorders where patients receive high-dose chemotherapy and/or radiation to eradicate the blood cancer and the remaining immune system, followed by the infusion of donor stem cells to reconstitute donor immunity (31). While tumor surveillance works through the graft-versus-tumor (GVT) effects of donor lymphocytes, dysregulated donor T cells can damage recipient target organs, leading to graft-versus-host disease (GVHD), the major complication of ASCT that occurs in 50%–80% of ASCT recipients (32). The systemic steroid is the treatment for GVHD but often increases life-threatening infections and blunts the GVT effects of donor T cells, causing the early relapse of blood cancer. Thus, a novel strategy is needed to maintain a fine balance between GVT and the anti-GVH effects of donor T cells in ASCT.

iNKT cells are thought to play a role in preventing GVHD in various preclinical studies (33–42). In clinical ASCT, a higher dose of iNKT cells in the donor grafts and the early recovery of iNKT cells after ASCT were associated with a decreased risk of GVHD (33–36). In murine ASCT, the activation of iNK T cells using Th2-polarizing αGalCer analogs or liposomal αGalCer eased murine GVHD, while Th1-polarizing αGalCer exacerbated it (37–39). More directly and applicable to clinical transplantation, the addition of purified CD4^+^ iNK T cells to the donor graft suppressed murine GVHD via the expansion of Tregs (40–42). Recently, *ex vivo* expanded human iNKT cells were shown to ameliorate GVHD in a xenogeneic GVHD model where lethally irradiated NOD-*scid* IL2Rg^null^ (NSG) mice received human peripheral blood mononuclear cells supplemented with human iNKT cells (11, 43, 44), supporting the feasibility of developing iNKT-cell-based therapy in ASCT. Thus, we evaluated the therapeutic potential of NKT10-enriched cord-derived iNKT cells in ASCT using a previously established xenogenic GVHD model.

## Materials and methods

### Human subjects

This study was performed in accordance with the research protocol approved by the University of Texas M.D. Anderson Cancer Center Institutional Review Committee and Institutional Biosafety Committee and the Declaration of Helsinki. Informed written consent from all study subjects was waived because the unidentifiable Leukopaks were obtained from a commercial provider (M.D. Anderson Blood Bank), and de-identified cord bloods allocated for general translational research were provided by M.D. Anderson Cord Blood Bank. Releasing identifiers is prohibited by established institutional regulations and policies per M.D. Anderson Cord Blood Bank.

### Sex as biological variables

There is no supporting evidence for differences in the phenotype and function of iNKT cells between genders. However, the age of donors and/or recipients may affect the phenotype and function of iNKT cells and GVHD outcome in ASCT (45, 46). Moreover, information on the age and sex of adult donors and cord units was not available as releasing identifying information is prohibited by institutional regulation at M.D. Anderson Blood Bank. In xenogenic GVHD experiments, we used 10–14-week-old female mice to eliminate confounding factors (gender and age) in evaluating xenogeneic GVHD.

### Animals

Animal experiments were conducted in compliance with the approved protocols of the MD Anderson Institutional Animal Care and Use Committee. There is no supporting evidence for differences in the phenotype and function of iNKT cells between genders; however, the age of donors and/or recipients may affect the phenotype and function of iNKT cells and graft-versus-host disease (GVHD) outcome in ASCT (45–47). Thus, we used 10–14-week-old female mice to eliminate confounding factors (gender, age) in evaluating xenogeneic GVHD. The 6-week-old NOD.Cg-Prkdc^SCID^IL2rg^tm1Wjl^/SzJ (NSG) mice were from Jackson Laboratories (Bar Harbor, Maine), acclimatized for three weeks before the experimental procedures, and maintained under specific-pathogen-free conditions. They were housed in micro-isolator cages and provided with acidified, antibiotic-treated water throughout the study.

### Materials

T-cell medium (TCM) was made of Roswell Park Memorial Institute Medium (RPMI)-1640 supplemented with L-glutamine (Gibco, #11875-093), 10% heat-inactivated fetal calf serum (FCS) (Hyclone, #A-1115-L), 0.001 μg/mL gentamicin (Gibco, #15710-015), 0.1 mM nonessential amino acids (Gibco, #11140-050) and essential amino acids (Gibco, #11130-051), 10 mM HEPES buffer solution (Gibco, #15630-080), and 5.5 μM 2-mercaptoethanol (2-ME) (Gibco, #21985-023). The freezing medium consists of 45% TCM, 45% FCS, and 10% dimethylsulfoxide (DMSO) (Sigma-Aldrich, #67-68-5). Phosphate-buffered saline (PBS, #10010-023) was from Gibco (Grand Island, NY, USA). Recombinant human IL-2 (#AF200-02) was from PeproTech (Cranbury, New Jersey). GM-CSF (#21-8339-CM01) and IL-4 (#21-8044-CM01) were from Tonbo Bioscience (San Diego, CA, USA). Alpha-galactosyl ceramide (αGalCer, #867000) was acquired from Avanti Polar Lipids (Alabaster, AL, USA) and solubilized in DMSO at concentrations ranging from 100 to 500 μM. Anti-iNKT microbeads (#130-094-842) and anti-CD3-microbeads (#130-050-101) were from Miltenyi Biotech (Gaithersburg, MD, USA). The following antibodies against specific targets were from BioLegend (San Diego, CA, USA), BD Bioscience (San Jose, CA, USA), or R&D systems (Minneapolis, MN, USA): iNKT-cell receptor (6B11), CD4 (RPA-T4), CD8α (SK11), CD25 (M-A251), CD56 (NCAM16.2), CD16 (3G8), CCR7 (3D12), CD161 (DX12), CD3 (OKT3), CD45RA (HI100), CD62L (DREG-56), ICOS (DX29), Lag3 (T47-530), HLA-DR (L243), CD11c (B-ly6), PD1 (BNI3), CD69 (FN50), CD127 (HIL-7R-M21), FoxP3 (PCH101), interferon (IFN)γ (B27), tumor necrosis factor (TNF)α (MAB11), IL-4 (8D4-8), IL-13 (JES10-5A2), IL-10 (JES3-9D7), Ki-67 (B56), and NRP1 (AD5-17F6, 12C2, and AF3870). Sheep IgG isotype control (#31243) was from Invitrogen, and GolgiStop protein transport inhibitor (#554724), GolgiPlug protein transport inhibitor (#555029), Fixable Viability Stain 620 (#564996), Cytometric Bead Array (CBA) Human Th1/Th2/Th17 cytokine kit (#560484), and BD Cytofix/Cytoperm Fixation/Permeabilization Solution Kit (#554714) were from BD Bioscience. FoxP3/Transcription Factor Staining Buffer set (#00-4423-00) was from eBiosience (San Diego, CA, USA). Ghost dye UV 450 (13-0868) was from Tonbo Bioscience (San Diego, CA, USA). The RNeasy Plus Micro Kit (#74034) and QIAamp DNA Blood Mini Kit (#51104) were from Qiagen (Germantown, MD, USA). CellTrace™ Carboxyfluorescein Succinimidyl Ester (CFSE) Cell Proliferation kit (#C34553) was from ThermoFisher Scientific (Carlsbad, CA, USA).

### *Ex vivo* analysis of iNKT cells

Peripheral blood mononuclear cells (PBMCs) and cord blood mononuclear cells (CBMCs) were isolated from Leukopaks or cord blood via density gradient centrifugation using Histopaque-1077 Hybri-Max (Sigma Life Science, #H8889). Due to the paucity of lymphocytes in CBMC, T cells from the entire CBMC from each cord blood unit were first enriched with magnetic activated cell sorting (MACS) with anti-CD3-microbeads according to the manufacturer’s guidelines, and all freshly enriched T cells were used for phenotypic analysis. Briefly, the freshly isolated PBMCs (5 × 10^6^) from adult donor or freshly enriched iNKT cells from CBMCs of each cord donor were stained with 100 μL of antibody cocktails in two sets, which contained the following antibodies at a concentration of 1 μg/mL in PBS: set 1 (CD3-FITC, CD8α-PerCP, CD4-APC, iNKTCR-PE, CD45RA-PB, CCR7-e780, CD62L-BV510, and CD127-PECy7) or set 2 (CD3-FITC, CD8α-PerCP, CD4-APC, iNKTCR-PE, CD56-PB, CD25-APC-Cy7, CD161-BV510, and CD16-PECy7). Subsequently, cells were acquired with BD FACSCanto II. FlowJo V10 (FlowJo LLC, Ashland, OR, USA) was used for the subsequent analysis. The iNKT cells from 46 consecutive adult donors and 45 consecutive cord units were assessed in 14 independent experiments.

### TCRβ repertoire analysis of freshly isolated iNKT cells from adult donor and cord blood

PBMCs (1 × 10^8^) and CBMCs (1 × 10^8^) were stained with CD3-FITC, iNKTCR-PE, CD4-APC, and Live/Death Fixable Aqua. The iNKT cells were sorted using BD FACSAria II Cell Sorter, and genomic DNA was isolated using the QIAamp DNA Blood Mini Kit. TCRβ repertoire was assessed by immunoSEQ hsTCRβ service (Adaptive Biotechnologies, Seattle, WA, USA) and analyzed with immunoSEQ Analyzer (Adaptive Biotechnologies).

### Preparation of allogeneic dendritic cells

Monocytes were isolated from PBMCs via a plastic adherence and cultured in TCM containing IL-4 (100 ng/mL) and GM-CSF (200 IU/mL) for 5 days as previously reported (12). The resulting allogeneic dendritic cells (allo-DCs) were cryopreserved for future use after irradiation using cesium-137 irradiator at a dose of 5, 000 cGу.

### *Ex vivo* isolation, stimulation, and expansion of iNKT cell from adult donors and cord blood

First, iNKT cells were enriched from PBMCs and CBMCs using MACS with anti-iNKT-microbeads according to the manufacturer’s guidelines, and then all of the iNKT cells were stimulated with 2 × 10^5^ allo-DCs in one well of a 24-well tissue culture plate in TCM containing αGalCer (100 nM) and IL-2 (200 IU/mL) for 14 days (12). Growth factors and TCM were replenished every 2 to 3 days, and iNKT cells were expanded into multiple wells if needed. At the end of the culture, iNKT cells were enumerated, and their quality (phenotype and function) was assessed before cryopreservation. For selected adult-derived iNKT cells, the CD4^+^ iNKT cells were further separated by MACS with anti-CD4 microbeads, and MACS-isolated CD4^+^ and CD4^-^ iNKT cells were subjected to the second antigenic stimulation. For selected cord-derived iNKT cells with suboptimal purity, iNKT cells were enriched again with MACS using anti-iNKTCR microbeads and expanded via a second antigenic stimulation.

### Flow cytometry

Up to 1 × 10^5^ iNKT cells were stained with antibody cocktail containing various antibodies at 1 μg/mL concentration and Fixable Viable Stain 620 or Ghost dye UV450 for 45 min at room temperature (RT) unless indicated otherwise. After washing, the cells were fixed with 2% paraformaldehyde (PFA) and acquired using an LSR Fortessa X-20 Cell Analyzer (BD Bioscience, Franklin Lakes, NJ, USA) or Cytek Aurora (Cytek Biosciences, Fremont, CA, USA). For intracellular staining of cytokines, cells were stained for surface markers as described above and fixed with 2% PFA or BD fixation/permeabilization buffer for 20 min, followed by washing. Subsequently, cells were permeabilized and stained with BD Perm/Wash buffer containing various anti-cytokine antibodies at 1 μg/mL for 45 min. After washing, cells were resuspended in PBS and subjected to acquisition. For FoxP3 and Ki-67 staining, surface-stained iNKT cells were fixed and permeabilized using the eBioscience FoxP3 transcription factor staining buffer containing anti-FoxP3 or Ki-67 antibodies at 1 μg/mL for 45 min. After washing, cells were acquired. Further analysis was performed using FlowJo version 10.3 (Tree Star, Ashland, OR, USA).

### T cell stimulation assay

For stimulation with phorbol 12-myristate 13-acetate (PMA) and Ionomycin, freshly expanded iNKT cells (1 × 10^5^ per well) were stimulated with TCM containing PMA (30 ng/mL), ionomycin (1 μg/mL), GolgiStop (1:1, 500 dilution), and GolgiPlug (1:1, 000 dilution) at 37 °C for 3 h in a 96-well plate. After staining for surface markers (CD3, iNKTCR, CD4, and CD8α), cells were assessed for intracellular cytokine production for the following cytokines: IFNγ, TNFα, IL-4, IL-10, and IL-13. The experiment was performed in quadruplicates.

For antigenic stimulation, previously cryopreserved iNKT cells were thawed in 37°C water bath for 5 min and then washed once in 10 nL of pre-warmed TCM. They were rested in TCM for 24 h prior to the experiment. The iNKT cells (1 × 10^5^ per well) were co-cultured with 50, 000 allo-DCs in the presence or absence of αGalCer (100 nM) at 37 °C for 72 h. To assess soluble cytokines, the culture supernatants were collected after 72 h of stimulation and assessed for the presence of various cytokines IL-2, IL-4, IL-6, IL-10, TNFα, IFNγ, and IL-17A using the BD CBA Human Th1/Th2/Th17 Cytokine kit as per the manufacturer’s guidelines. The experiment was performed in triplicates or quadruplicates.

### RNA-seq

The freshly expanded iNKT cells from adult donor and cord blood at resting status were stained for the following surface markers: CD3, iNKTCR, CD4, and Live/Dead Fixable 620, followed by FACS-sorting for CD4^+^iNKTCR^+^CD3^+^ or CD4^-^iNKTCR^+^CD3^+^ cells from adult-derived iNKT cells and CD4^+^iNKTCR^+^CD3^+^ from cord-derived iNKT cells. Total RNA was extracted from adult (CD4^+^ and CD4^-^) and cord iNKT cells using the RNeasy Plus Micro Kit, following the manufacturer’s protocol. Subsequent assessment of total RNAs for both quantity and integrity were conducted using an RNA 6000 Nano chip on a 2100 BioAnalyzer (Agilent; Santa Clara, CA, USA), yielding an average RNA integrity score of 8.6. Subsequent RNA library preparation and sequencing were performed at Avera Institute for Human Genetics (AIHG) (Sioux Falls, SD) with HiSeq2500 (Illumina, Inc, San Diego, CA, USA), producing paired-end 100-bp reads, with an average of 53 million paired-end reads per sample.

The resulting sequence read data were processed and converted to FASTQ format for subsequent analysis using the Illumina BaseSpace analysis software, specifically FASTQ Generation v1.0.0.

### RNA-seq data analysis

Raw FASTQ sequences were assessed for quality control using FastQC (version 0.11.5), followed by adapter removal. The trimmed reads were mapped to the CRCh38/hg38 reference genome using TopHat2 (version 2.0.14). Samtools (version 1.2) was used to sort, convert between formats, and calculate mapping statistics. Gene annotation was carried out using the GENCODE annotation (version 25), and aligned reads were summarized at the gene level using HTseq-count. R (3.5.1) and Bioconductor package DESeq2 were used to identify differentially expressed genes as follows: First, the read count was pre-filtered to remove genes expressed at extremely low levels. Then, DESeq2 was carried out for read count filtering, normalization, dispersion estimation, and identification of differential expression. DESeq2 modeled the counts using a negative binomial distribution, followed by the Wald test. The final *p*-value was adjusted using the Benjamini and Hochberg method. Principal component analysis (PCA) was conducted using DESeq2 on variance-stabilizing transformed data from differentially expressed genes across the three pairwise comparisons (cord-derived iNKT cells, adult-derived CD4^+^ iNKT cells, and adult-derived CD4^-^ iNKT cells). The three-group-heatmap was defined by combining a balanced number of the most significant differentially expressed genes from three pairwise comparisons between the three groups (cord-derived iNKT cells, adult-derived CD4^-^ iNKT cells, and adult-derived CD4^+^ iNKT cells).

### Single-cell RNA-seq/TCRseq

The freshly expanded adult- and cord-derived iNKT cells were stimulated by αGalCer-pulsed allogeneic DCs for 24 h and stained for CD3 and iNKTCR. Live iNKT cells were then sorted using BD FACSAria II Cell Sorter (BD Biosciences, San Jose, CA, USA). Then, the cells were processed through the 10X Genomics Chromium Next GEM Single Cell 5’ V2 chemistry with TCR enrichment amplification following the manufacturer’s protocol (CG000331). Briefly, the cells were loaded onto a Chromium Next GEM Chip K (10X Genomics PN#1000286) at the recommended concentration to capture 7, 000 to 10, 000 cells along with Single Cell 5’ Gel Beads containing barcoded oligos. Using the Chromium Controller, thousands of cells were partitioned into nanoliter-scale gel bead-in-emulsion (GEM) droplets for single-cell reactions. Within the droplets, the cells were lysed and the cellular RNAs were barcoded using reverse transcriptase from the poly(dT) RT primers, resulting in full-length cDNA from poly-adenylated mRNA. Barcoded RNAs were released, and the pool of single-cell libraries was amplified and cleaned up. One portion was used for the full-length TCR capture using TCR enrichment, and the second portion was used for RNA library construction. The TCR region was selected using TCR primers using the Chromium Single-Cell Human TCR Amplification Kit (10X Genomics PN#1000252), and NGS libraries were constructed. The other portion of the amplified full-length mRNA library was used to construct 5’ RNA libraries using Chromium Next GEM Single Cell 5’ Reagent Kit v2 (10X Genomics PN#1000263).

### scRNA-seq analysis

The raw scRNA-seq data were demultiplexed, pre-processed, and aligned to the human genome (hg38) using CellRanger 3.1 (10X Genomics). The resulting expression matrices were imported into R, and Seurat version 5.0.3. was used for the subsequent downstream analysis. The quality controls excluded doublets and cells with fewer than 250 genes or 500 features or high mitochondrial content (>10%) or low ribosomal content (<10%). After the filtered read matrix was log-normalized, the samples were combined, and the potential batch effects were corrected using the Harmony R package. To visualize the data effectively, further dimensionality reduction was performed using uniform manifold approximation and projection (UMAP), and differential expression analysis was performed after excluding *TRA* and *TRB* genes using the FindAllMarkers function. Pseudotime analysis was performed starting from cluster 2 or 8 using the Monocle3 package. The scTCR-sequencing data were seamlessly integrated with single-cell RNA sequencing data within Cell Ranger (10x), and further analysis for expansion-contraction patterns, gene correlations, and clonotype dynamics was performed using scRepertoire V2.0.

### Ingenuity Pathway Analysis

Ingenuity Pathway Analysis (IPA) version 122103623 (Qiagen, Aarhus, Denmark) was used to identify pathways predicted to be activated or inhibited on cord-derived iNKT cells compared with adult-derived iNKT cells (reference) with differential gene expression based on RNA-seq data or cluster 2 compared with cluster 8 (reference) with those based on scRNA-seq data, using parameters including a log2 fold change threshold of -2 for downregulated genes and 2 for upregulated genes, with the adjusted *p*-value set at 0.01.

### T cell suppression assay

Freshly isolated PBMCs (1 × 10^7^/mL in PBS) were labeled with CSFE at 1 μM for 30 min at 37 °C according to the manufacturer’s instruction, and unlabeled CSFE was quenched with TCM. CSFE-labeled PBMCs at 5 × 10^5^ per well in a 96-well plate were stimulated with anti-CD3 and anti-CD28 antibodies at 1 ug/mL for 16 h, followed by washing. The iNKT cells were added at various ratios of PBMCs to iNKT cells and cultured for an additional 5 days. Cells were stained for CD3, iNKTCR, CD4, and CD8α, and CSFE^+^T cells were assessed by flow cytometry. % proliferation: (CSFE^int/low^ T cells)/CSFE^+/-^ T cells × 100. % inhibition of proliferation: (% proliferation of T cells (control group) - % proliferation of T cells (experimental group))/(% proliferation of T cells (control)) × 100. For mixed lymphocyte reaction, T cells from PBMC were activated by soluble anti-CD3/CD28 antibodies (1 ug/mL) and expanded for 6 days, followed by cryopreservation for future use. These memory T cells (1 × 10^5^/well) were thawed and rested for 1 day before the experiment. Subsequently, 1 × 10^5^ T cells were placed in one well of a 96-well plate and co-cultured with allo-DCs (1 × 10^4^/well) with or without iNKT cells (5 × 10^3^) in the presence of soluble anti-CD3 antibody at 0.1 μg/mL. After 5 days of culture, T cells were stained for CD3, iNKTCR, CD4, CD8α, Ki-67, and NRP1, and their expression was assessed by flow cytometry. The experiment was performed in quadruplicate.

### Xenogeneic murine GVHD model

NSG mice were irradiated with a single dose of 200 cGy using a ^137^Cs irradiator on day -1 and received 1 × 10^7^ iNKT cell^depleted^ PBMCs with or without 5 × 10^5^ adult-derived CD4^+^ or CD4^-^ iNKT cells or cord-derived iNKT cells at a dose of 5 × 10^5^, 1 × 10^5^, and 2 × 10^4^ on day 0. Subsequently, the mice were monitored for survival and clinical GVHD assessed by weight loss (0 = <10%, 1 = 10%–25%, 2 = >25%), posture (0 = normal, 1 = hunching at rest, 2 = severe hunching), activity (0 = normal, 1 = mild decrease, 2 = stationary unless stimulated), fur texture (0 = normal, 1 = moderate ruffling, 2 = severe ruffling), and skin condition (0 = normal, 1 = scaling or mild hair loss, 2 = presence of denuded skin) as previously outlined (48, 49). Moribund mice were euthanized in accordance with institutional guidelines. A subset of mice was sacrificed on day 14 after infusion, and the liver was procured and processed for routine histopathological analysis and evaluation by a pathologist.

### Statistical analysis

GraphPad Prism version 10.00 (GraphPad, San Diego, CA, USA) was used for the statistical analysis. Paired Student’s *t*-test was used to compare values between paired CD4^+^ or CD4^-^ iNKT cells derived from the same adult donor. Unpaired Student’s *t*-test was used to compare values among adult-derived CD4^+^ or adult-derived CD4^-^ iNKT cells vs. cord-derived iNKT cells or between conditions within the same group. Survival curves were plotted using the Kaplan–Meier method, and differences in survival rates among groups were assessed using the log-rank (Mantel–Cox) survival test. A *p*-value less than 0.05 was deemed statistically significant and noted with an asterisk symbol (*) unless otherwise indicated.

## Results

### CD4^+^CD25^+^CD161^low^ iNKT cells are enriched in cord blood

Age is a contributing factor for the phenotypic and functional heterogeneity of iNKT cells in healthy individuals, as older individuals reportedly have a lower frequency of iNKT cells with increased CD4^+^ subtype, Th2-biased cytokine production, and decreased expansion capacity compared with young individuals (46, 50, 51). Interestingly, iNKT cells from the thymus and cord blood consist of predominantly the CD4^+^ subtype with a higher expression of CD25 than those from adult donors (29, 30), suggesting that CD4^-^ iNKT cells may undergo robust peripheral expansion early in life, leading to phenotypic alteration with age. CD4^+^ vs. CD4^-^ phenotype of iNKT cells is thought to correlate with the Th2 vs. Th1-functional polarization upon activation, respectively (7–9, 11, 12). Thus, iNKT cells from cord blood may display a unique Th2-biased, regulatory function given the predominant presence of the CD4^+^ subtype. To explore the utility of cord blood iNKT cells as a candidate for novel cell therapy to promote immune-regulation, we first investigated the phenotype and TCR usage of iNKT cells from cord blood compared with those from adult donors.

**Figure 1:**
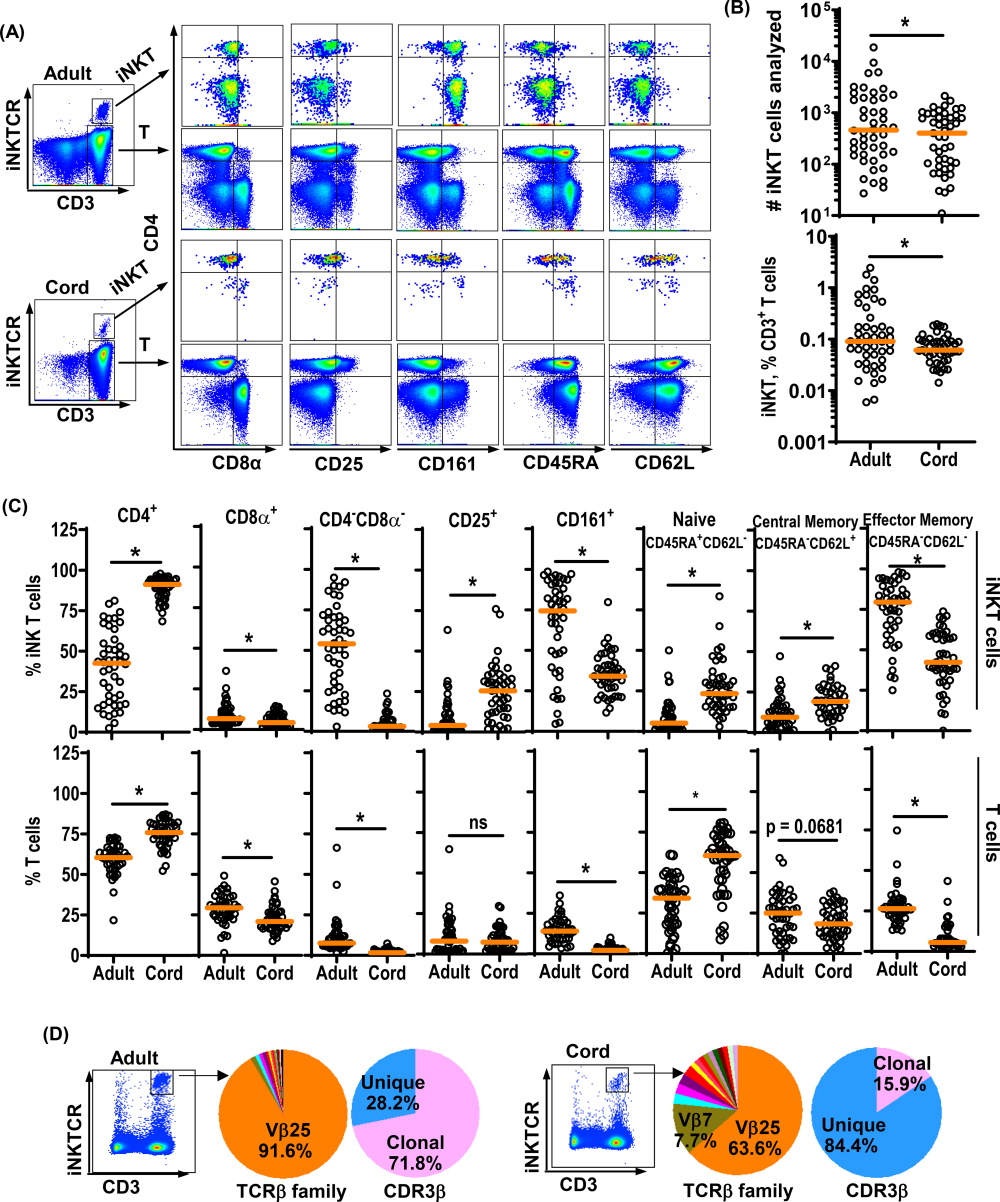
*Ex vivo* analysis of iNKT cells from adult donor and cord blood. **(A)** Representative flow cytometry analysis of various surface markers on *ex vivo* isolated iNKT cells and conventional T cells from 46 consecutive adult donor and 45 consecutive cord blood. **(B)** Percentage of iNKT cells of CD3^+^T cells and absolute numbers of iNKT cells analyzed for phenotype analysis. **(C)** Percent CD4^+^, CD8α^+^, CD4^-^CD8α^-^, CD25^+^, CD161^+^, naïve phenotype (CD45RA^+^CD62L^+^), central memory (CD45RA^-^CD62L^+^), and effector memory (CD45RA^-^CD62L^-^) iNKT cells (top) and T cells (bottom). **(D)** TCRβ repertoire analysis of *ex vivo* isolated iNKT cells from adult and cord donor. First, average of 1, 631 and 547 iNKT cells from adult and cord donor, respectively, were subjected for further phenotype analysis. The iNKT cells in cord were present at a median 0.061% of T cells (range: 0.014 to 0.944) with predominantly CD4^+^ subtype (median 91%, range: 68.4% to 97.8%). They were enriched with CD4^+^CD25^+^CD161^low^, naïve or CM phenotype. On the contrary, iNKT cells from adult donors were present at a median 0.090% of T cells (range: 0.005 to 2.432) with varied frequencies of CD4^+^ subtype (median: 42.6%, range 2.4% to 78.2%) and CD4^-^CD8^-^ subtypes (median: 54.0%, range 2.8% o 92.4%). They were enriched with CD161^+^ and EM phenotype. Lastly, cord iNKT cells utilized diverse TCRVβ repertoire (Vβ25 63.6%, unique TCRCDR3β 84.4%) than adult iNKT cells (Vβ25 91.6%, unique TCRCDR3β 23.2%). A single symbol represents a value from a single donor. Unpaired Student’s *t*-test was used to compare the differences between adult and cord groups.

One challenge in assessing iNKT cells from cord blood is the relative rarity of T cells from CBMCs compared with PBMCs from adult donors. To ensure high-quality data from an adequate number of iNKT cells for phenotypic analysis, we first enriched CD3^+^ T cells from all CBMCs isolated from each cord unit using MACS with anti-CD3-microbeads, and the entire population of enriched T cells was subjected to a subsequent flow cytometric analysis. The median number of iNKT cells analyzed was 400 (range: 11–2, 116) from 45 consecutive cord blood units and 459 (range: 27–18, 670) from 46 consecutive adult donors. The median frequency of iNKT cells from cord blood was 0.061% (range: 0.014%–0.194%), which was significantly lower than that from adult donors –0.090% (range: 0.005%–2.438%, *p* = 0.035) (**Figures 1A, B**). As expected from previous studies (29, 30), iNKT cells from cord donors were predominantly CD4^+^ subset (median 91.4%, range: 68.4% to 97.8%) and significantly enriched with CD25^+^ or CD161^low^ subtype and naïve (CD45RA^+^CD62L^-^) and central memory (CD45RA^+^CD62L^+^) phenotype, whereas those from adult donors showed a median of 42.6% CD4 subsets with large donor-to-donor variation (range: 2.4% to 81.2%) and were enriched with CD25^-^ or CD161^+^ subtype and effector memory (CD45RA^-^CD62L^-^) phenotype (**Figures 1A–C**). Lastly, iNKT cells from cord blood used a diverse TCRβ repertoire compared to those from adult donors. Only 63.6% of cord iNKTCRα were paired with the *TRB25* family and 84.4% of iNKTCRβ showed unique CDR3β, whereas 91.9% of adult iNKTCRα were paired with the *TRB25* family and only 28.2% of adult iNKTCRβ used unique CDR3β (**Figure 1D**).

### iNKT cells from cord blood undergo robust expansion via single antigenic stimulation and maintain their enrichment with CD4^+^CD25^+^FoxP3^+^ subtype

Previously, we demonstrated that a single antigenic stimulation using αGalCer-pulsed allo-DCs led to the robust expansion of *ex vivo* isolated iNKT cells from PBMCs to a clinically meaningful number with high purity (11, 12). Here we evaluated whether one can consistently obtain highly pure iNKT cells from 13 consecutive cord blood units using a single antigenic stimulation.

**Figure 2:**
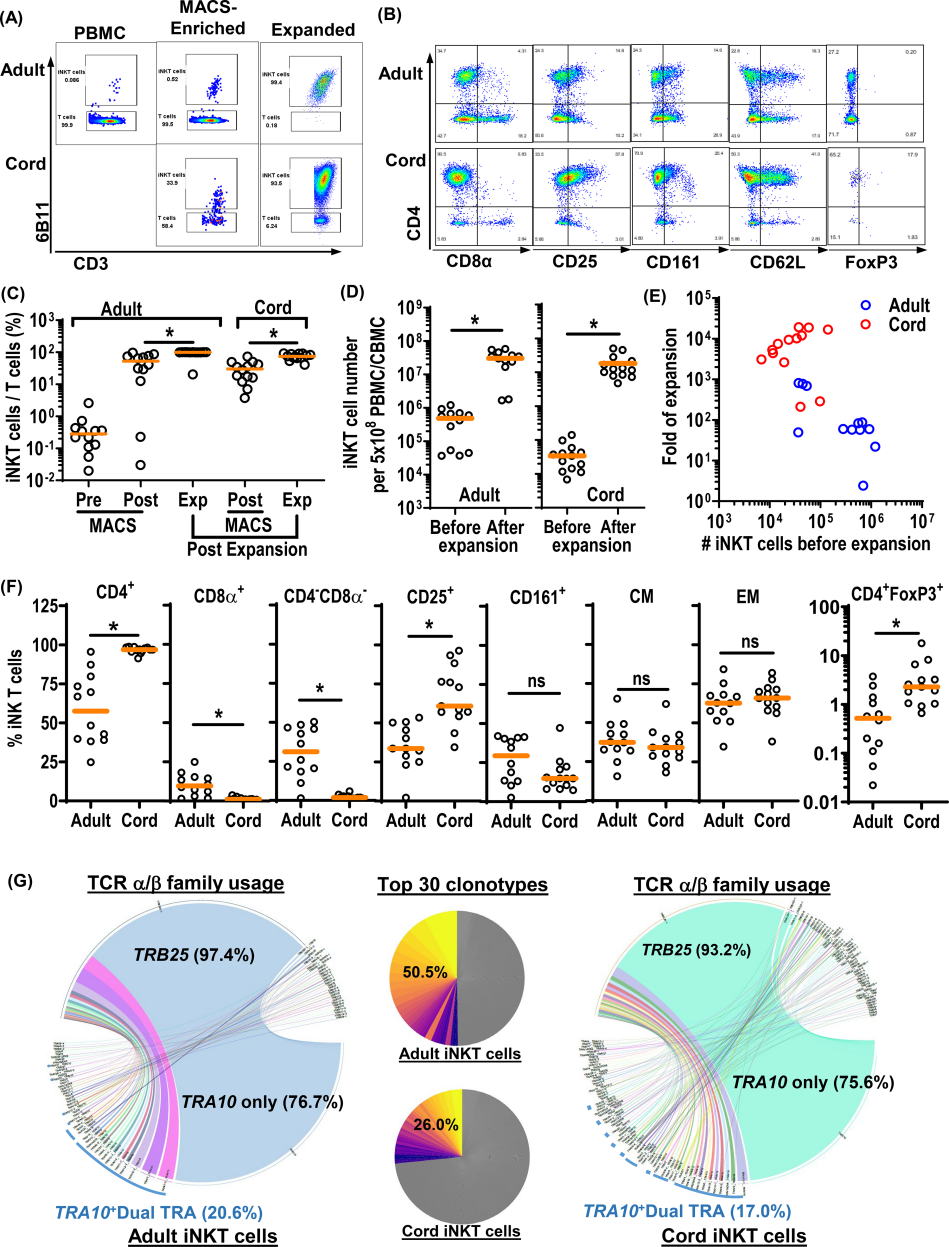
Phenotypic characterization of adult- and cord-derived iNKT cells. The magnetically sorted iNKT cells from 12 consecutive peripheral blood mononuclear cells (PBMC) of adult donors and 13 consecutive cord blood mononuclear cells (CBMC) of cord blood donors were expanded with a single antigenic stimulation with αGalCer pulsed allogeneic DCs in IL-2 for 2 weeks and assessed for quality, quantity, and phenotype. **(A)** Flow cytometric analysis of iNKT cells from selected adult and cord donor prior to and after MACS enrichment and expansion. **(B)** Flow cytometric analysis of various surface markers and FoxP3 from selected adult- and cord-derived iNKT cells. **(C)** iNKT cell purity of iNKT cells from adult and cord donors prior to and after MACS-enrichment and antigenic expansion. **(D)** Absolute numbers of iNKT cells from 5 × 10^8^ PBMC of adult donors or 5 × 10^8^ CBMC of cord donors prior to and after expansion. **(E)** Fold of expansion of adult- and cord-derived iNKT cells plotted against the absolute number of iNKT cells isolated from 5 × 10^8^ PBMC or CBMC prior to expansion. **(F)** Percentages of CD4^+^, CD8α^+^, CD4^-^CD8α^-^, CD4^+^CD25^+^, CD161^+^, central memory (CD45RA^-^CD62L^+^), effector memory (CD45RA^-^CD62L^-^), and CD4^+^FoxP3^+^ phenotypes of adult- and cord-derived iNKT cells. **(G)** Single-cell TCRα/β repertoire analysis of adult- and cord-derived iNKT cells. The iNKT cells were effectively enriched with MACS isolation and expanded, resulting to an average of 92.9% and 74.2% iNKT cells from adult and cord donors, respectively. Although the absolute number of iNKT cells isolated from cord donors was lower than those from adult donors, 4.13 × 10^4^ vs. 4.58 × 10^5^, respectively, but the cord iNKT cells underwent an average of 8, 409 folds of expansion, leading to 1.83 × 10^7^ iNKT cells, while the adult iNKT cells underwent an average of 229 folds of expansion, leading to 3.03 × 10^7^ iNKT cells. Cord-derived iNKT cells were exclusively CD4^+^ and enriched with CD25^+^FoxP3^+^ phenotype, while adult-derived iNKT cells contained varying degrees of CD4^+^ phenotype and significantly higher CD8α^+^ and CD8α^-^CD4^-^ subtypes than cord-derived iNKT cells. Both adult- and cord-derived iNKT cells utilized exclusively Vβ25 family (adult donor: 97.4% vs. cord donor: 93.2%) and Vα10 family (adult donor: 97.3% vs. cord donor: 92.6%); however, adult-derived iNKT cells contained a larger portion of the top 30 most common clonotypes (50.5%) compared to cord-derived iNKT cells (26.0%). A symbol represents a value from a single donor. Paired Student’s *t*-test was used to assess differences of iNKT purity and numbers among pre- and post-MACS-enrichment and expansion **(C, D)**, and unpaired Student’s *t*-test was used to assess differences in iNKT phenotype between adult and cord donors **(F)**.

First, the median frequency of iNKT cells substantially increased to 28.8% after MACS enrichment and to 75.2% after a single antigenic stimulation (**Figures 2A, C**). Although the purity of cord-derived iNKT cells was lower than those from 12 consecutive adult donors (median 99.9%), the purity of cord-derived iNKT cells could reach up to 99% after the second antigenic stimulation (data not shown). As expected from the lower T cell content in CBMCs, the absolute number of cord iNKT cells before the antigenic expansion was lower than that from adult iNKT cells (median 34, 080 vs. 486, 309; *p* = 0.0007). However, cord iNKT cells underwent significantly higher folds of expansion than adult iNKT cells (median 7, 520 vs. 59.4-fold change, *p* = 0.003), reaching 1.2 × 10^7^ iNKT cells, which was not significantly different from the final amount of iNKT cells derived from adult donors (3.2 × 10^7^, *p* = 0.071) (**Figures 2D, E**).

Cord-derived iNKT cells were exclusively of the CD4^+^ subset compared with adult-derived iNKT cells (96.9%, range: 91.3%–98.8% vs. 57.5%, range: 24.8%–70.7%, *p* < 0.0001) with significant enrichment of CD25^+^ (60.8%, range: 34.5%–96.2% vs. 33.6%, range: 2.2% to 53.3%, *p* = 0.0413) and CD4^+^FoxP3^+^ subtype (2.28%, range: 0.66%–17.9% vs. 0.53%, range: 0.02% to 3.73%, *p* = 0.0413) (**Figures 2B, F**). The Sc-TCRseq analysis of cord-derived iNKT cells revealed that iNKT cells with *TRB25* were preferentially expanded after antigenic stimulation (93.2% vs. 63.6%, after vs. before expansion) but continued to have a diverse TCRβ repertoire. The top 30 clonotypes of iNKTCR from cord-derived iNKT cells comprised only 26% of total iNKTCR compared with 50.5% of those from adult-derived iNKT cells (**Figure 2G**; **Supplementary Table 1**). Lastly, 17% of cord-derived iNKT cells expressed a second TCR alpha chain in addition to *TRA10*, suggesting that iNKT cells may have dual antigen specificity.

### Cord-derived iNKT cells display a Th-2/10-biased cytokine profile

The ability to produce Th1 and/or Th2-type cytokines is one mechanism by which iNKT cells can influence adaptive immunity in the immune-microenvironment (10, 52), and this functional plasticity arises in part from the Th1- or Th2-biased cytokine production profile of CD4^-^ and CD4^+^ iNKT subsets, respectively (7–9, 11, 12). Thus, we postulated that cord-derived iNKT cells display a Th2-biased cytokine production profile given the predominant presence of CD4^+^ iNKT cells compared with adult-derived iNKT cells.

**Figure 3:**
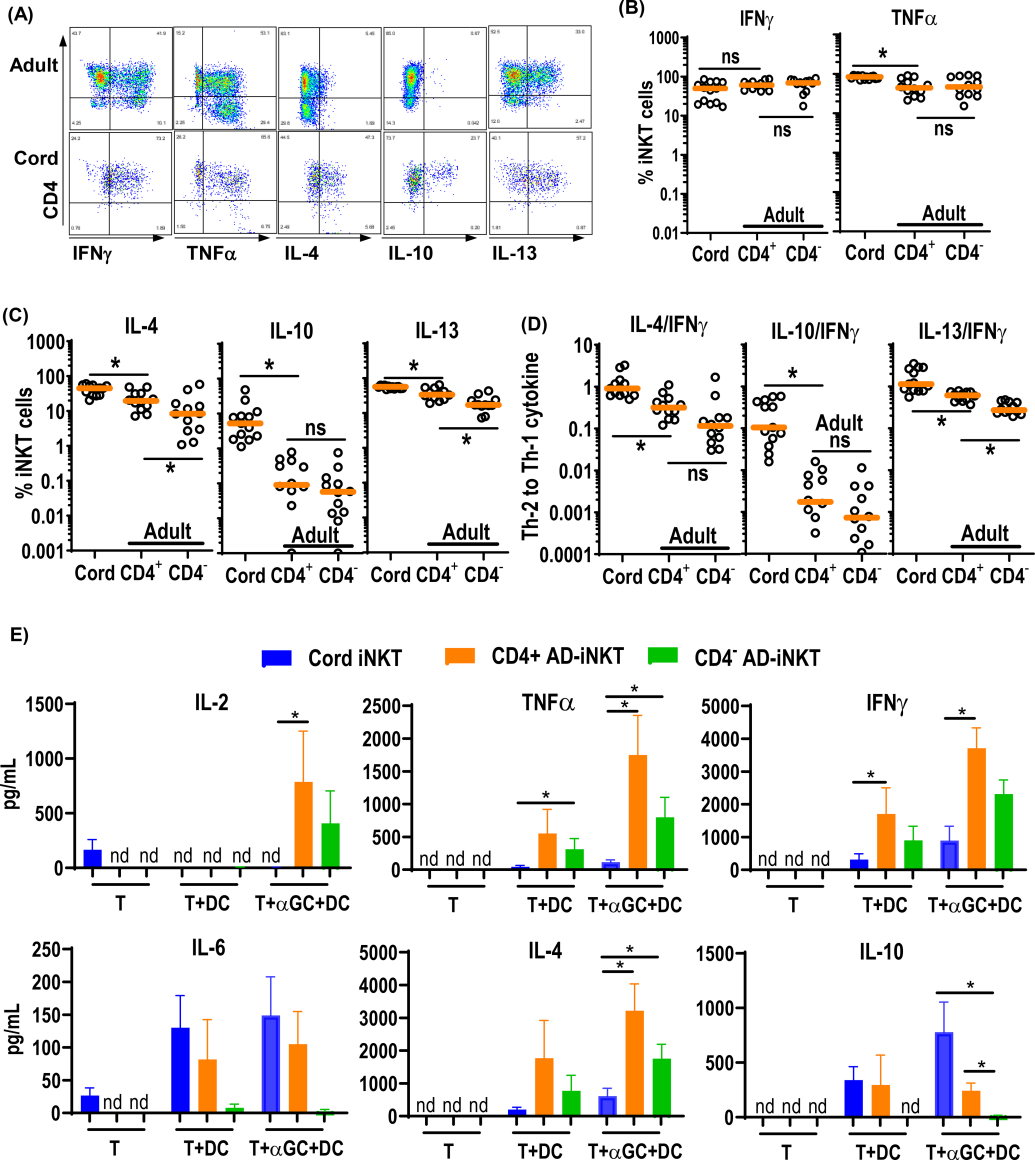
Functional characterization of adult- and cord-derived iNKT cells. The iNKT cells were stimulated with phorbol myristate acetate (PMA) and Ionomycin, and intracellular cytokine production was assessed. **(A)** Representative flow cytometric analysis of intracellular cytokine production of activated iNKT cells from adult and cord donors. **(B)** Percentage Th1 cytokine (IFNγ and TNFα)^+^ and **(C)** Th2 cytokine (IL-4, IL-10, and IL-13)^+^ iNKT cells from cord donors and CD4^+^ or CD4^-^ iNKT cells from adult donors. **(D)** Ratios of Th-2 cytokine (IL-4, IL-10, and IL-13)^+^ to Th-1 cytokine (IFNγ)^+^ iNKT cells from adult and cord donors. The iNKT cells were stimulated with αGalCer pulsed DCs for 72 h, and cytokine in the culture supernatant was assessed. **(E)** Soluble cytokine production by paired CD4^+^ or CD4^-^ iNKT cells from five adult donors or iNKT cells from 10 cord donors after stimulation with DCs pulsed with αGalCer. Upon stimulation with PMA, cord-derived iNKT cells contained significantly higher Th2 cytokine (IL-4, IL-10, and IL-13)^+^ iNKT cells compared to adult-derived CD4^+^ or CD4^-^ iNKT cells, leading to a Th2-biased cytokine production profile. Upon antigenic stimulation, cord-derived iNKT cells produced soluble IL-10 significantly higher than adult-derived iNKT cells, but IFNγ or TNFα in lower levels, confirming Th2/10-biased cytokine production profile. A symbol represents a value from a single donor. Paired Student’s *t*-test was employed to compare differences between CD4^+^ vs. CD4^-^ iNKT cells derived from the same adult donor, and unpaired Student’s *t*-test was used to assess differences between adult- and cord-derived iNKT cells. *P*-value less than 0.05 was indicated as significant. *p < 0.05; NS, not significant; ND, not detected.

To elucidate the intrinsic differences in Th1- vs. Th2-type cytokine production between adult-derived CD4^+^ or CD4^-^ and cord-derived iNKT cells, we assessed an array of Th1-type (TNFα and IFNγ) and Th2-type cytokines (IL-4, IL-10, and IL-13) in iNKT cells after activation with PMA/Ionomycin. Cord-derived iNKT cells produced IFNγ at a similar level to that of adult-derived iNKT cells but produced TNFα better than adult-derived CD4^+^ iNKT cells (83.9% vs. 44.2%, *p* = 0.002, **Figures 3A, B**). As expected, cord-derived iNKT cells produced all Th2-type cytokines—IL-4, IL-10, and IL-13—significantly better than adult-derived CD4^+^ iNKT cells, leading to higher ratios of Th2-type cytokines (IL-4, IL-10, and IL-13) to Th1-type cytokine (IFNγ, **Figures 3A, C, D**). Notably, a large portion of cord-derived iNKT cells (median: 5.224%, range: 1.13 to 48.6%) produced IL-10, a potent immunoregulatory cytokine, whereas both adult-derived CD4^+^ and adult-derived CD4^-^ iNKT cells barely expressed IL-10.

To confirm the intrinsic Th2/10-biased cytokine production profile of cord-derived iNKT cells, we assessed the ability of cord-derived iNKT cells to produce soluble cytokines upon antigenic stimulation using αGalCer pulsed allo-DCs and compared with the ability of adult-derived CD4^+^ or CD4^-^ iNKT cells to produce soluble cytokines. Unlike what we observed with activation with PMA/ionomycine, cord-derived iNKT cells preferentially produced IL-10 at significantly higher amounts than adult-derived CD4^+^ or CD4^-^ iNKT cells upon antigenic stimulation, whereas cord-derived iNKT cells produced significantly lower amounts of both IFNγ and TNFα than adult-derived iNKT cells (**Figure 3E**). Interestingly, IL-6, a pleomorphic cytokine, was produced by both cord-derived and adult-derived CD4^+^ iNKT cells, suggesting that IL-6 may be a CD4 subset- specific cytokine.

### Cord-derived iNKT cells exhibit a distinct gene expression signature associated with NKT10 cells and multiple immunoregulatory signaling pathways

To explore the potential regulatory properties of cord-derived iNKT cells compared with adult-derived iNKT cells, we investigated the transcriptional landscape from freshly expanded, cord-derived iNKT cells from six cord blood units and paired CD4^+^ and CD4^-^ iNKT cells derived from six adult donors at the resting stage using RNA-seq analysis.

**Figure 4:**
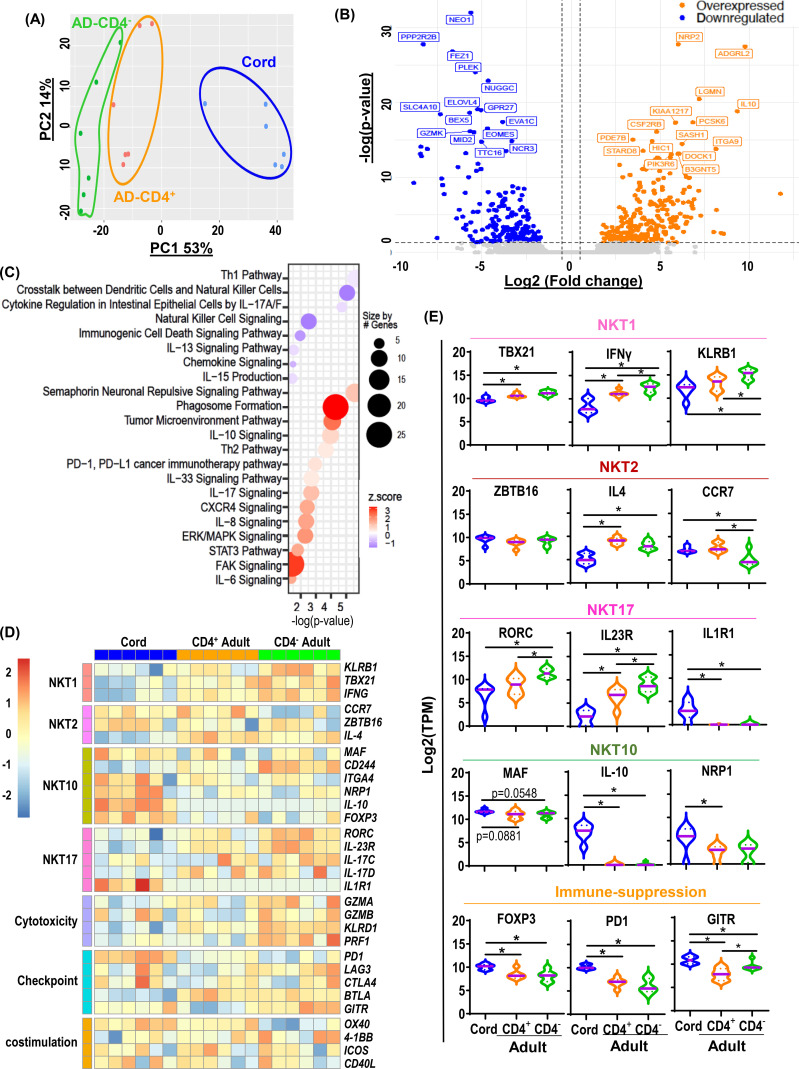
Cord-derived iNKT cells display a distinct gene expression profiling favoring immune-regulation. CD4^+^ and CD4^-^ adult-derived iNKT cells or cord iNKT cells were purified with FACS and subjected to RNA-seq analysis. **(A)** Principal component analysis (PCA) of gene expression from CD4^+^ and CD4^-^ adult-derived iNKT cells or cord-derived iNKT cells. **(B)** A volcano plot illustrates the fold change in gene expression [log2(fold change)] against statistical significance [log10(p-value)]. The differential gene expression between cord vs. adult iNKT cells (reference) was evaluated using DESeq2. Genes with an FDR-adjusted *p*-value below 0.05 are shaded gray, while upregulated and downregulated genes are highlighted in orange and blue, respectively. **(C)** Ingenuity Pathway Analysis demonstrating the upregulated (red) or downregulated (purple) immune pathways in cord-derived iNKT cells compared to adult-derived iNKT cells. **(D)** Heatmap displaying gene expression profiles from distinct iNKT cell subsets and other relevant surface molecules, including NKT1, NKT2, NKT10, NKT17, cytotoxic markers, immune checkpoints, and costimulatory molecules in both adult (CD4^+^ and CD4^-^) and cord iNKT cells. **(E)** Selected gene expression associated with NKT1, NKT2, NKT10, NKT17, and immune-suppression. Violin plot depicting transcript per million (TPM) values on a log2 scale, with the median indicated by a horizontal line. Cord-derived iNKT cells display a distinct gene expression profile compared to CD4^+^ or CD4^-^ or combined adult-derived iNKT cells, enriched with genes associated with NKT10, immune-checkpoint proteins, and pathways related to immune-regulations. Unpaired Student’s *t*-test was used to compare the differences between adult- and cord-derived iNKT cells, and paired Student’s *t*-test was used to assess the differences between CD4^+^ or CD4^-^ adult-derived iNKT cells from the same donor. The *p*-value is indicated where appropriate.

First, principal component analysis (PCA) of the differential expression of 14, 447 genes revealed a segregation of cord-derived iNKT cells from adult-derived iNKT cells, with cord-derived cells being the most distinct from adult-derived CD4^-^ iNKT cells (**Figure 4A**; **Supplementary Figure 1**). Differential gene expression analysis identified 365 genes that were significantly upregulated or downregulated in cord-derived iNKT cells compared with adult-derived iNKT cells (**Figure 4B**; **Supplementary Table 2**), and IL-10 was among the top five most upregulated genes in cord-derived iNKT cells, confirming the presence of IL-10-producing iNKT cells (NKT10 subset) in cord-derived iNKT cells at a relatively higher abundance than in adult-derived iNKT cells.

Moreover, ingenuity pathway analysis predicted that multiple immunoregulatory pathways such as IL-10 signaling, Th2 pathways, and PD-1/PD-L1 cancer immunotherapy pathways were upregulated in cord-derived iNKT cells, whereas several pathways related to the iNKT effector functions such as Th1 pathways, NK cell signaling, IL17A/F signaling, and chemokine signaling were upregulated in adult-derived iNKT cells (**Figure 4C**; **Supplementary Table 3**).

Lastly, we evaluated the expression of genes associated with murine iNKT functional subsets to assess the relative abundance of each functional subset in human iNKT cells (**Figures 4D, E**) (10, 13, 14, 28). As expected, genes associated with NKT10 cells (*MAF*, *ITGA4*, *NRP1*, *IL-10*, and *FoxP3*) and immune-suppression (*PD1*, *GITR*, and *CTLA4*) were enriched in cord-derived iNKT cells. In contrast, genes associated with effector functions such as NKT1 (*KLRB1*, *TBX21*, and *IFN*γ), NKT17 (*RORC*, *IL-23R*, and *IL-17C/*D), and cytotoxicity (*GZMA/B*, *KLRD1*, and *PRF1*) were upregulated in adult-derived CD4^-^ iNKT cells. The use of costimulatory molecules was dichotomized in that cord-derived iNKT cells were enriched with Th2/9-promoting OX40, whereas adult-derived iNKT cells used 4-1BB in potentiating effector function (53–55). Lastly, cord-derived iNKT cells expressed *IL-1R1* (**Figures 4D, E**) and *IL-1A* (data not shown), suggesting the potential to differentiate into NKT17 via IL-1β (56, 57).

Thus, our findings indicate that cord-derived iNKT cells are enriched with a gene expression signature associated with IL-10-producing NKT10 cells and multiple immunoregulatory pathways.

### scRNA-seq analysis of cord-derived iNKT cells identifies human NKT10 cells

The immunoregulatory role of IL-10-producing iNKT cells, NKT10 cells, has been demonstrated in murine models of autoimmune diseases, metabolic disorders, and tumor surveillance (15–18, 25–27). More recently, human iNKT cells isolated from the lamina propria of patients with Crohn’s disease were shown to produce IL-10 in variable levels, which correlated with lower frequencies of pathogenic Th17 cells (27). However, the phenotypic and functional characterization of human NKT10 cells and their role in the pathogenesis of human diseases has not been well studied, likely due to the paucity of human NKT10 cells. Because the cord-derived iNKT cells contained high frequencies of IL-10-producing iNKT cells (**Figure 3C**, average 9.75% ± 13.0%, *n* = 13 donors), we aimed to delineate NKT10 cells from other NKT functional subsets via scRNA-seq analysis of human iNKT cells freshly expanded from adult donors and cord blood units and activated with αGalCer-pulsed DCs.

**Figure 5:**
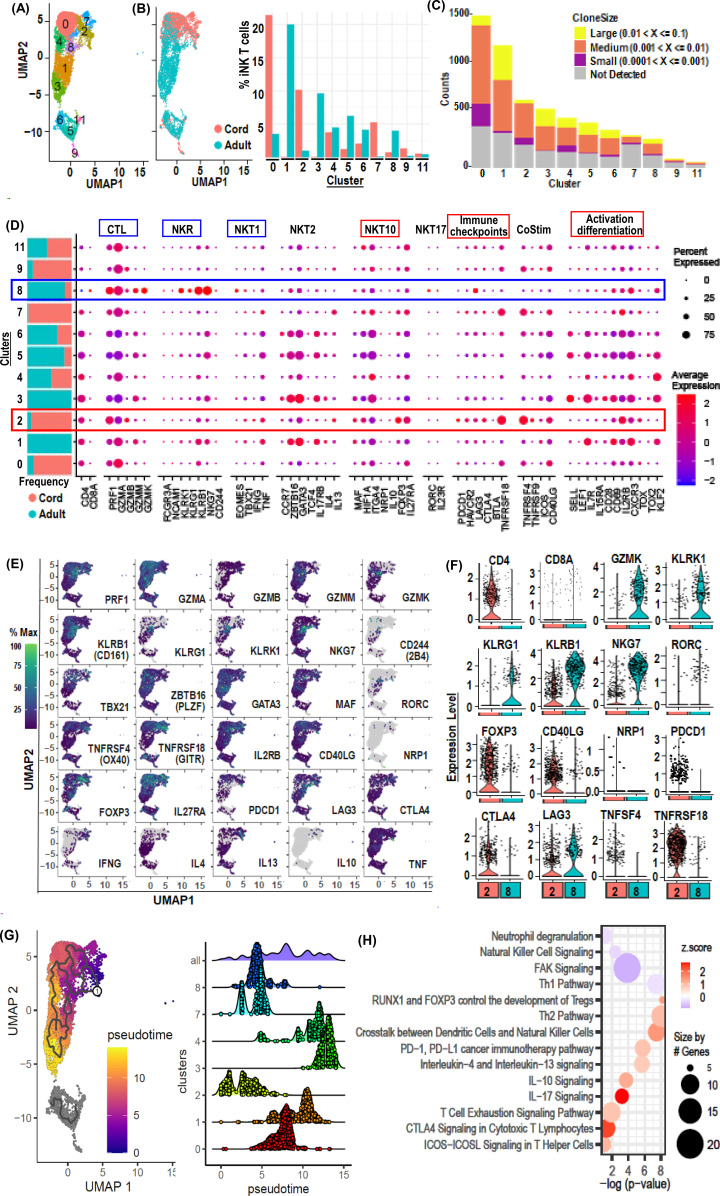
Single-cell analysis identifies NKT10-like subset enriched in cord-derived iNKT cells. FACS-purified adult or cord-derived iNKT cells were subjected to single-cell RNA/TCRseq. **(A)** Uniform manifold approximation and projection (UMAP) plots of single-cell RNA expression from combined adult and cord iNKT cells identified 11 functional clusters. **(B)** Distribution of adult or cord iNKT cells in UMAP plot and within each cluster. **(C)** Distribution of TCR clonality within each cluster. **(D)** Dot plot visualization of gene expression associated with CTLs, NKRs, NKT1, NKT2, NKT10, NKT17, immune checkpoints, costimulation, and activation and differentiation per cluster. **(E)** Density plot of selected gene expression. **(F)** Feature violin plots of selected gene expressions of cluster 2 vs. cluster 8. **(G)** Pseudotime analysis starting at a node within cluster 2 or cluster 8. **(H)** Ingenuity Pathway Analysis of significantly upregulated or downregulated immune-related pathways of cluster 2 over cluster 8. The single-cell gene expression analysis of combined adult- and cord-derived iNKT cells identified 11 functional clusters. Cord-derived iNKT cells were enriched in cluster 0 and 2, while adult-derived iNKT cells were dominant in 3, 5, 6, and 8. Cluster 2 has upregulated genes associated with NKT10 and immune-checkpoint proteins, while cluster 8 displays a high expression of CTL granules and NKR. Accordingly, cluster 2 showed upregulated pathways related to immune-regulation over cluster 8.

First, uniform manifold approximation and projection of differential gene expression of adult-derived and cord-derived iNKT cells identified 11 functional clusters, and cluster 10 was excluded from subsequent analysis because it contained a B-cell-specific signature (**Figure 5A**; **Supplementary Figure 2**; **Supplementary Table 4**). The remaining 10 clusters were formed into two groups: clusters 0, 1, 2, 4, 7, and 8 and clusters 5, 6, 9, and 11. Clusters 5, 6, 9, and 11 were enriched with genes associated with the mitosis of cells such as *TUBB*, *TYMS*, *CENPA*, *AURKB*, etc., and represented cells under active division, whereas clusters 0, 1, 2, 5, 7, and 8 represented cells at resting status. Cord-derived iNKT cells were dominant in clusters 0, 2, and 7, and adult-derived iNKT cells were mostly present in clusters 1, 3, and 8. Cluster 4 contained both cord-derived and adult-derived iNKT cells (**Figure 5B**). Adult-derived iNKT cells (clusters 1, 3, and 8) had a higher fraction of large clone size compared with cord-derived iNKT cells (clusters 0, 2, and 7), suggesting that cord-derived iNKT cells used a diverse TCR repertoire compared with adult-derived iNKT cells, consistent with *ex vivo* analysis of the TCR repertoire of cord iNKT cells (**Figures 1D**, **5C**).

Differential gene expression analysis revealed two distinct clusters—cluster 2 with an immunoregulatory gene signature (NKT10) highlighting the overexpression of *TNFRSF18* (*GITR*), *TNFRSF4* (OX40), and *FOXP3* vs. cluster 8 with a cytotoxic effector gene signature (NKT1/17) consisting of *NKG7*, *GZMK*, *KLRK1* (NKG2D), *KLRB1* (CD161, NKR-P1A), *EOMES*, and *RORC* (**Supplementary Figure 2**; **Figures 5D–F**). Clusters 0 and 7, dominant with cord-derived iNKT cells, displayed an immunoregulatory gene signature similar to that of cluster 2, although it was not as distinct. Clusters 1, 3, 5, and 6, dominant with adult-derived iNKT cells, showed the overexpression of NKT2-associated genes such as *ZBTB16* (PLZF), *GATA3*, *IL17RB*, and *IL-4*. All clusters expressed cytotoxic granules (PRF1 and GZMA) to varying degrees, supporting the overlapping effector–regulator functions of human iNKT cells.

Pseudotime analysis showed a closer association of cluster 2 (NKT10) to cluster 8 (NKT1/17) than cluster 1 or 3 (NKT2) (**Figure 5G**). Immune-related pathway analysis of cluster 2 (NKT10) over cluster 8 (NKT1/17) confirmed the upregulation of multiple immune-regulatory pathways related to Treg, Th2, PD1-PD-L1, CTLA4, and IL-10 signaling (**Figure 5H**; **Supplementary Table 5**). The IL-17 signaling pathway was upregulated in cluster 2 (NKT10), suggesting a possible functional transition between cluster 2 (NKT10) and cluster 8 (NKT1/17) and vice versa. Moreover, 11% of the TCR clonotypes were shared between clusters 2 and 8, supporting potential trans-differentiation between NKT10 and NKT1/17 (**Supplementary Figure 4**).

To validate the presence of two distinctive functional clusters—NKT10 (NRP1^+^ PD1^+^ GITR^+^OX40^+^ CD40L^+^) and effector NKT1/17 (NKG2D^+^CD161^+^)—identified from the scRNA-seq analysis of iNKT cells from one adult donor and one cord blood unit, we evaluated iNKT cells derived from 10 adult donors and 10 cord blood units for the surface expression of selected markers for the effector or regulator phenotype via multi-dimensional flow cytometry (**Supplementary Figure 4**). Dimensional reduction of 14 analysis parameters using the Crusty with Pleograph algorithm (58) showed the functional heterogeneity of iNKT cells consisting of 12 distinct functional subsets, but the clusters were segregated into two groups: group 1 with clusters 10, 4, 8, 7, and 6, enriched with effector markers (CD16/CD56, NKG2D, and CD161), and group 2 with clusters 11, 9, 1, 5, and 12, enriched with regulator markers (NRP1, LAG3, TIM3, PD1, CTLA4, and CD357). In particular, cluster 12 most resembled the regulator subset, whereas cluster 10 was similar to the effector subset identified from the scRNA-seq analysis (**Supplementary Figure 4**).

### Cord-derived human iNKT cells exert *in vitro* and *in vivo* immunosuppressive function

**Figure 6:**
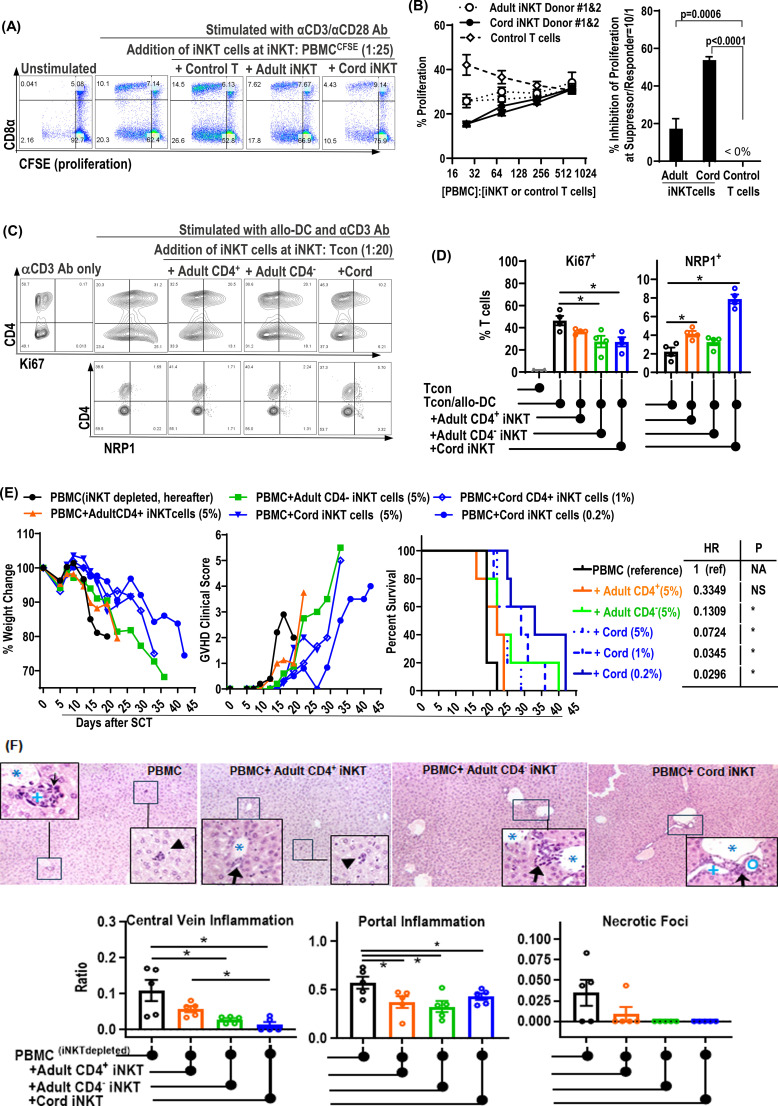
Cord-derived iNKT cells display *in vitro* and *in vivo* immunosuppressive function better than adult iNKT cells. Representative flow cytometry analysis of activated CSFE^+^ T cells **(A)** and their percent proliferation **(B)** at 5 days after co-culture with iNKT or control T cells at various ratios of PBMC to iNKT cells. Representative flow cytometry analysis of NRP1 and Ki-67 of T cells **(C)** and their percent NRP1 or K67 expression **(D)** at 5 days after co-culture with allogeneic DCs in the presence of absence of iNKT cells and anti-CD3 antibody. The experiments were performed in quadruplicate, and one of two independent experiments is shown. **(E)** Sub-lethally irradiated NSG mice received iNKT-depleted PBMC with or without CD4^+^ or CD4^-^ adult-derived NKT cells or cord-derived iNKT cells at different ratios of PBMCs to iNKT cells and monitored for clinical GVHD and survival. **(F)** Hematoxylin and eosin (H&E)-stained liver from mice at 2 weeks post-infusion of PBMC with or without CD4^+^ or CD4^-^ adult-derived iNKT cells or cord-derived iNKT cells. Portal inflammation (arrow) and necrotic foci (arrow head) were noted. *, central vein; O, central artery; +, bile duct. The results from one of the two independent experiments are shown. Each symbol represents a value from a single mouse. Overall survival was plotted using Kaplan–Meier survival curve, and differences in survival among groups was assessed using log-rank test. Unpaired Student’s *t*-test was used to compare differences in pathological findings between groups. *P*-value less than 0.05 was considered significant. *p < 0.05. Cord-derived iNKT cells suppressed the proliferation of conventional T cells when stimulated by anti-CD3/CD28 antibodies or by allogeneic DCs plus sub-optimal anti-CD3 stimulation, better than CD4^+^ or CD4^-^ adult-derived iNKT cells, and in part via induction of NRP1 on conventional T cells. In addition, cord-derived iNKT cells ameliorated clinical xenogeneic GVHD better than adult-derived iNKT cells when supplemented to the donor graft in a dose-dependent manner and significantly reduced inflammation in central and portal veins as well as necrotic foci in the liver.

The iNKT cells are thought to have a beneficial role in preventing GVHD in ASCT (34, 35, 40–42, 59), and *ex vivo* expanded human iNKT cells have been shown to mitigate the xenogenic GVHD (11, 43, 44), suggesting human iNKT cells as potential cell therapeutics in ASCT. Because cord-derived iNKT cells are enriched with IL-10-producing NRP1^+^ NKT10 cells, they may confer better immunosuppressive activity than adult-derived iNKT cells (**Figures 3**-**6**). Here we investigated the therapeutic potential of cord-derived iNKT cells to promote immune-regulation in xenogenic graft versus host disease model.

To evaluate the suppressive function *in vitro*, iNKT cells were co-cultured with conventional T cells stimulated with antibodies against CD3/CD28 in various ratios of PBMC to iNKT cells, and the degree of T cell proliferation was measured (**Figures 6A, B**). As anticipated, cord-derived iNKT cells, not control T cells, significantly suppressed the proliferation of activated T cells better than adult-derived iNKT cells in a dose-dependent manner. However, T cells are fully activated by a combination of CD3/CD28 stimulation, and this may not represent the activity of alloreactive T cells that occur during ASCT.

To evaluate the suppressive function of iNKT cells against alloreactive T cells, we performed modified mixed lymphocyte reaction where memory T cells were stimulated by allogeneic DCs and a suboptimal dose of anti-CD3 antibody in the presence or absence of iNKT cells at a ratio of 20 T cells to one iNKT cell (**Figures 6C, D**). Here we used the incorporation of Ki-67 as a marker of T cell proliferation as this would be more sensitive than measuring the dilution of CFSE (**Figures 6A, B**). The allogeneic T cells proliferated only in the presence of allogeneic DCs, and the addition of cord-derived iNKT cells significantly decreased the proliferation of alloreactive T cells, similar to adult-derived iNKT cells. However, cord-derived iNKT cells led to the upregulation of NRP1 on alloreactive T cells significantly more than adult-derived iNKT cells (**Figure 6D**), suggesting the additional mechanism of immune-regulation.

Furthermore, we investigated whether cord-derived iNKT cells ameliorate xenograft graft-versus-host disease (GVHD) in a murine model (**Figures 6E, F**). Here lethally irradiated NSG mice received a donor graft consisting of iNKT-depleted PBMCs with or without cord-derived iNKT cells at various ratios of iNKT cells to PBMCs (1:20, 1:100, and 1:500) and were monitored for clinical signs of GVHD and survival, as previously reported (11). In this xenogeneic GVHD model, NSG mice developed severe GVHD, characterized by rapid body weight loss and other clinical signs of GVHD (ruffled fur, hunched posture, decreased activity, skin lesions, and rapid weight loss) as well as pathologic signs of damage in the liver (central vein and portal inflammation as well as necrosis), which resulted in 100% mortality by day 22, with a median survival of 19 days. As expected, mice that received donor graft supplemented with cord-derived iNKT cells underwent an improved clinical and pathologic course of xenogeneic GVHD and survival (hazard ratio (HR) = 0.0724, *p* < 0.05) better than mice that received donor graft supplemented with adult-derived CD4^+^ iNKT cells (HR = 0.3349, not significant) or adult-derived CD4^-^ iNKT cells (HR = 0.1309, *p* < 0.05). Interestingly, the anti-GVH effects of cord-derived iNKT cells were inversely correlated to the percentage of iNKT cells within the donor graft: HR 0.0724 for 5%, 0.0345 for 1%, and 0.0296 for 0.2%.

## Discussion

IL-10-producing iNKT cells, NKT10 cells, constitute a distinct regulatory subset of iNKT cells and are enriched in the CD4^+^ subset of iNKT cells that overexpress certain immunosuppressive molecules such as NRP1, GITR, CTLA-4, and PD1 and transcription factors associated with cMAF (15, 28). Murine NKT10 cells are known to mediate immune-regulation in murine models of obesity (16–18), diabetes (17, 18, 25), multiple sclerosis (15, 26), tumor progression (15), and colitis (27). Unlike other murine iNKT subsets such as NKT1, NKT2, and NKT17 which follow thymic developmental pathways (14, 60), murine NKT10 cells appear to emerge through extrathymic development after antigenic stimulation or during inflammation (15). In humans, IL-10-producing iNKT cells were reportedly present in very low frequencies in healthy donors but could expand via antigenic stimulation in the presence of TGFβ (15, 61). Moreover, human iNKT cells isolated from the lamina propria of patients with Crohn’s disease were shown to produce IL-10 in variable levels, which correlated with lower frequencies of pathogenic Th17 cells, supporting the potential immunosuppressive role of human NKT10 cells in the pathogenesis of inflammatory bowel diseases (27). However, the phenotype, transcriptional landscape, and function of human NKT10 cells are vastly unknown, likely due to the relative paucity of the NKT10 subset in human iNKT cells. Therefore, we performed a comprehensive analysis of human NKT10 cells present in polyclonal iNKT cells derived from cord blood and adult donors via single antigenic stimulation (11, 12) and confirmed the presence of IL-10-producing human iNKT cells, which were present at significantly higher frequencies in cord blood (average 9.754%, *N* = 13 consecutive donors) than in adult donors (average 0.1772%, *N* = 12 consecutive donors) with a clear Th2/10-biased cytokine production profile (**Figure 3**).

Analysis of the gene expression profiles of NKT10-enriched cord-derived NKT cells compared with adult-derived iNKT cells revealed that human NKT10 cells may have a unique transcriptional landscape distinct from murine NKT10 cells. Although NKT10-enriched, cord-derived iNKT cells and NKT10-like cluster 2 showed a gene expression signature similar to that of murine NKT10 cells and upregulated immunoregulatory pathways involving Tregs, IL-10 signaling, PD1-PDL1 axis, and Th2 pathways, both cord-derived iNKT cells and NKT10-like cluster 2 showed upregulated pathways associated with IL-17 signaling, suggesting that human NKT10 cells may be closely related to human NKT17 populations (**Figures 4**, **5**). Moreover, NKT10-like cluster 2 shared a substantial portion of the TCR clonotypes (11%) with NKT1/17-like cluster 8 (**Supplementary Figure 3**), indicating a possible transition between the human NKT10 and NKT17 subsets. This functional plasticity of human iNKT subsets is a novel finding and distinct from that of murine iNKT functional subsets whose transcriptional framework is highly divergent (13, 14, 28).

The ability of iNKT cells to produce Th-2/10-type cytokines is essential to promote immune-tolerance in adaptive immunity by suppressing Th-1-induced inflammation and by activating suppressor immune cells, including myeloid-derived dendritic cells (MDSC), tolerogenic DCs and macrophages, and Tregs (16–18, 26, 27, 59, 62–64). Although Th-2/10-biased iNKT responses can be driven by the presence of NKT2/10 subsets, for example, in cord-derived iNKT cells, the manner in which iNKT cells are stimulated appears to be critical in driving Th-1- or Th-2-biased iNKT responses. First, the structural modification of the agonist glycolipid antigen αGalCer can polarize iNKT function toward Th-1- or Th-2-biased responses (65–68), and these Th-2-biasing αGalCer analogues have been shown to ameliorate several autoimmune diseases in preclinical murine models, demonstrating the therapeutic potentials of iNKT antigen modulators (37, 69–72). Second, human iNKT cells preferentially produce Th-2 type cytokines when stimulated by αGalCer presented by non-professional antigen-presenting cells (APCs) such as Schwann cells or by weak agonist or self-glycolipid antigens presented by professional APCs, suggesting that autoreactive iNKT cells may primarily play a role in immune-regulation (6, 11). Interestingly, cord-derived iNKT cells preferentially produced IL-10 with significantly less IFNγ or TNFα compared with adult-derived iNKT cells when stimulated by allogeneic DCs in the absence of αGalCer (**Figure 3E**), supporting the potential therapeutic utility of cord-derived iNKT cells as immune-regulators.

The immune-adjuvant ability of iNKT cells has been extensively investigated and exploited to augment anti-tumor immunity by activating iNKT cells with αGalCer or αGalCer-loaded DCs (19–24). Furthermore, iNKT-cell-based adoptive cell therapy has utilized intrinsic anti-tumor activity to treat metastatic melanoma (73) or enhanced anti-tumor and pro-inflammatory properties with chimeric antigen receptor (CAR) to treat medulloblastoma or CD19^+^ B cell malignancies in a preclinical and clinical study, respectively (23, 74). Recently, the regulatory property of off-the-shelf, *ex vivo* expanded human iNKT cells has been investigated to treat COVID-19-related acute respiratory distress syndrome with promising results (75). However, there is still a gap in the clinical translation of a potent regulatory function of iNKT cells into therapeutics to treat various immune-related disorders likely due to the profound functional heterogeneity and relative paucity of regulatory subsets in human iNKT cells. Not only did we demonstrate that single antigenic stimulation can lead to a robust expansion of iNKT cells from cord blood to a clinically meaningful number in extremely high purity but also we showed that cord-derived iNKT cells contained significantly higher frequencies of NKT10 subsets with unique gene expression signatures in multiple immunoregulatory pathways. Furthermore, we showed that these NKT10-enriched cord-derived iNKT cells displayed superior *in vitro* and *in vivo* immunosuppressive function in a xenogeneic GVHD murine model, supporting the therapeutic potential of NKT10-enriched cord-derived iNKT cells as a third-party cell therapy to mitigate pathologic inflammation in a variety of human immune disorders (**Figure 6**).

Allogeneic stem cell transplantation (ASCT) is one such example where adoptive cellular therapy with NKT10-enriched cord-derived iNKT cells could be supplemented to donor graft to prevent GVHD. Moreover, human iNKT cells derived from cord blood have several advantages over conventional Tregs to prevent GVHD in transplantation. First, cord-derived iNKT cells can be used in off-the-shelf fashion to prevent and treat GVHD because they are easily obtained from HLA-compatible cord blood units for ASCT recipients. Second, cord-derived iNKT cells may have additional anti-leukemic effects (GVL) via direct iNKTCR-mediated cytolysis and NK-like effector function (76). Third, cord-derived iNKT cells can enhance anti-microbial and tumor T-cell immunity of the donor graft through the CD40–CD40L feedback loop with DCs. Lastly, the preserved GVL effects of cord-derived iNKT cells can be re-directed to specific tumor antigens via CAR, and these CAR CB-iNKT cells can be a novel platform for off-the-shelf CAR-T cells with an improved safety profile due to intrinsic Th-2-type cytokine production (76). Thus, *ex vivo* expanded NKT10-enriched cord-derived iNKT cells are excellent candidates for an innovative cell therapy to improve the outcome of ASCT.

## Data Availability

The datasets presented in this study can be found in online repositories. The names of the repository/repositories and accession number(s) can be found in the article/**Supplementary Material**.
