# Supplementary material for: Human NKT10 cells are enriched in cord-derived invariant natural killer T cells and mediate immune-regulation in a xenogeneic graft-versus-host disease model
Source: Front Immunol. 2026 Jun 10;17:1834739. doi: 10.3389/fimmu.2026.1834739 (PMC13291024; doi:10.3389/fimmu.2026.1834739)
Supplement: Supplementary file 1 [file DataSheet1.docx]

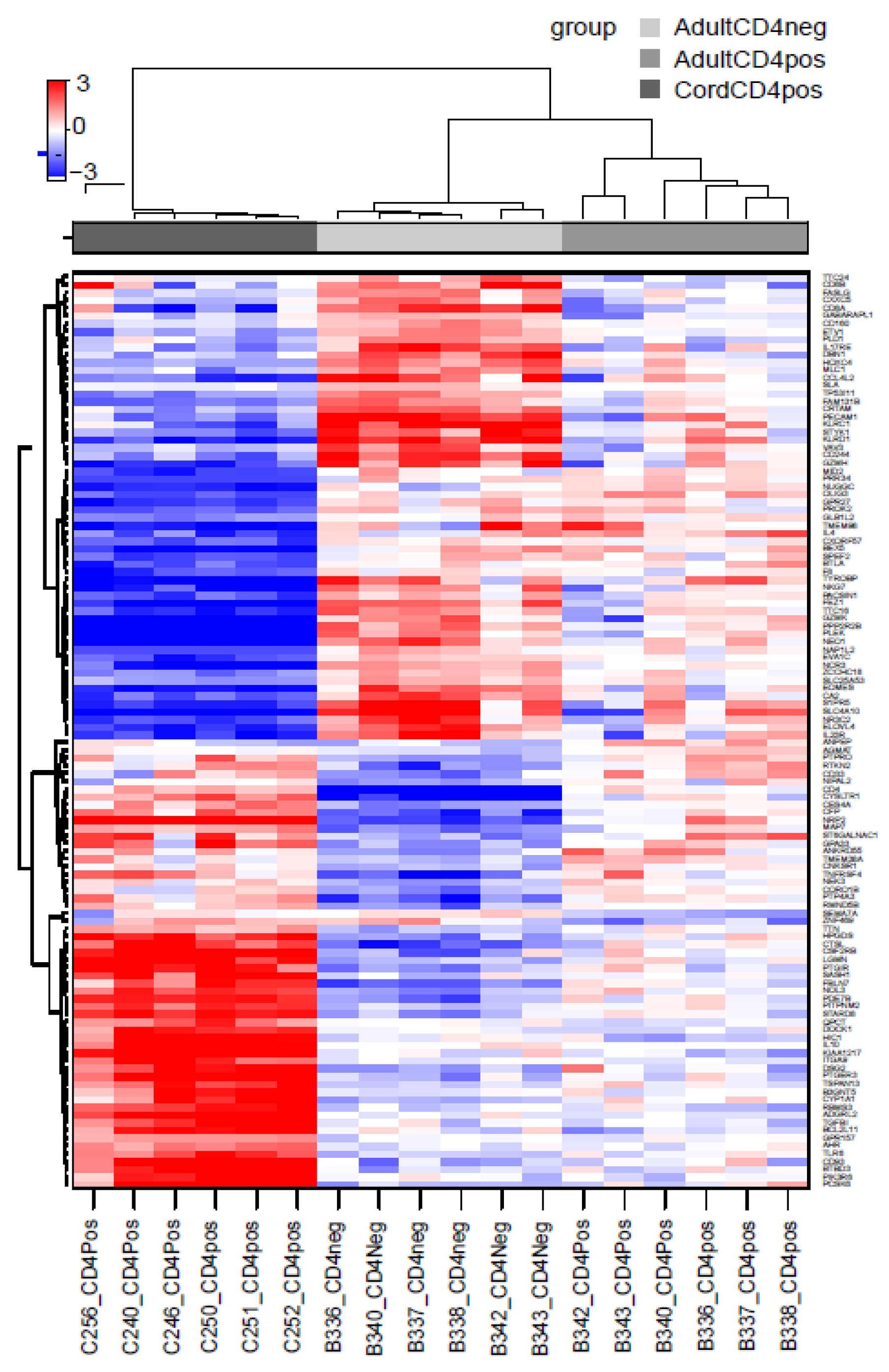
Supplementary Figure 1. Differentially expressed genes among cord iNKT cells (6 cord blood donors) vs paired CD4^+^ and CD4^-^ iNKT cells (6 adult donors).


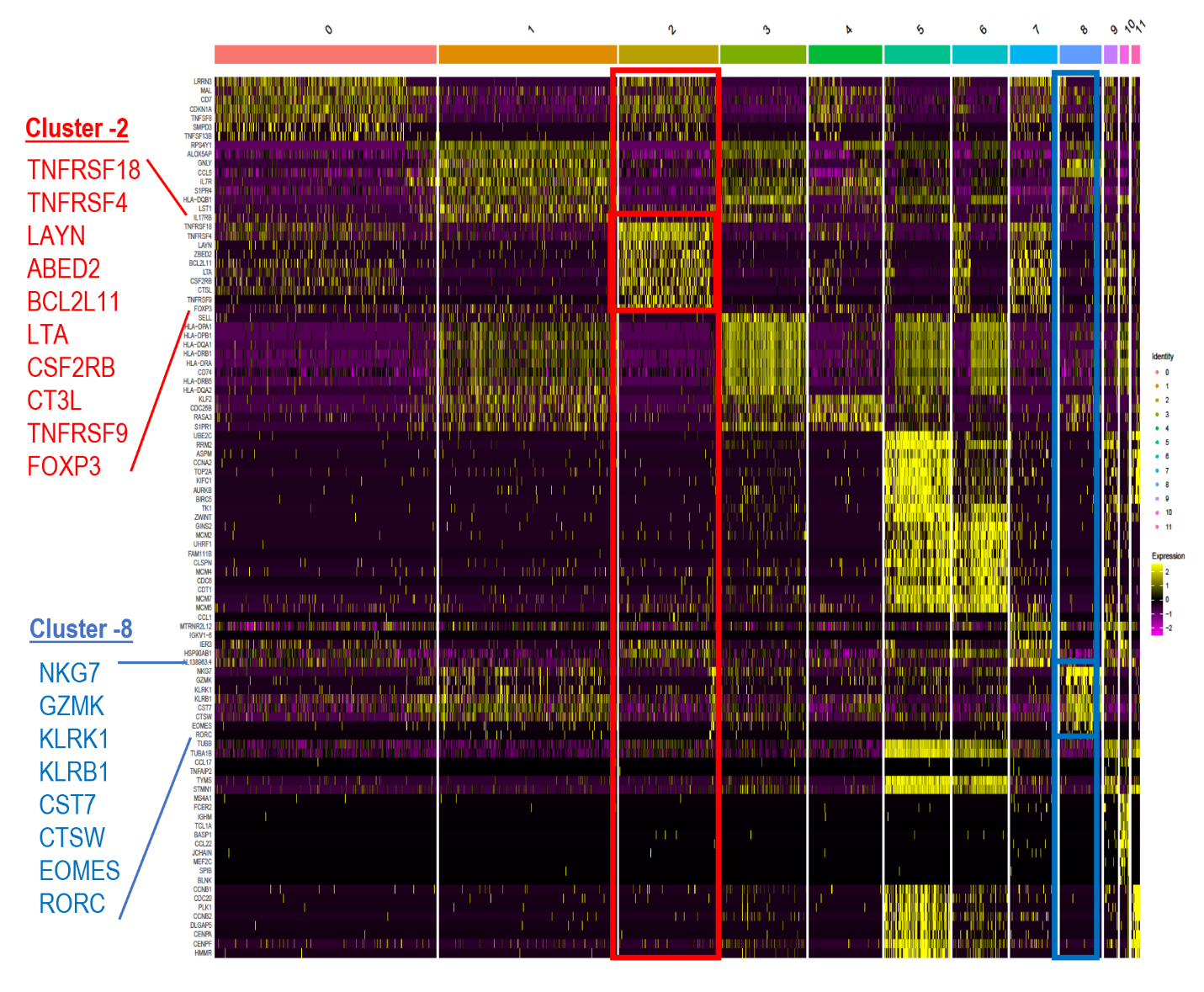


Supplementary Figure 2. Differentially expressed genes among clusters projected from single cell RNAseq analysis of iNKT cells from 1 cord blood and 1 adult donor. Cluster 2 is enriched with genes associated with gene related to immune-regulation such as TNFRSF1 (GITR), TNFRSF4 (OX40), TNFRS9 (4-1BB), and FOXP3, while cluster 8 upregulated genes associated with cytotoxicity and effector T cells such as NKG7, GZMK (granzyme K), KLRK1 (NKG2D), KLRB1 (CD161), EOMES, and RORC.


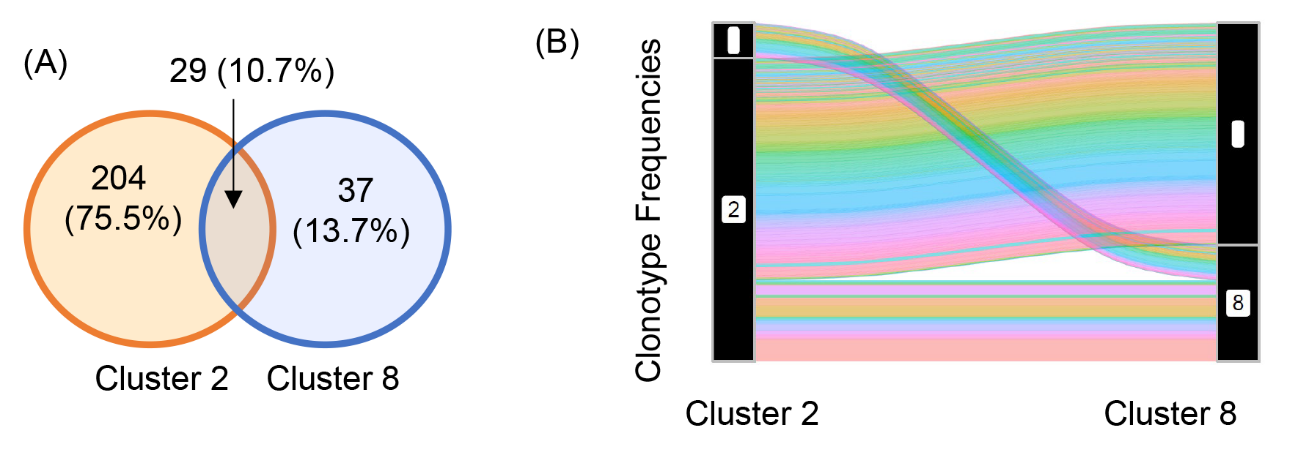


Supplementary Figure 3. The T cell receptor usage (Clonotype) of cluster 2 and 8 represented with venn diagram (A) and Alluvial analysis (B).


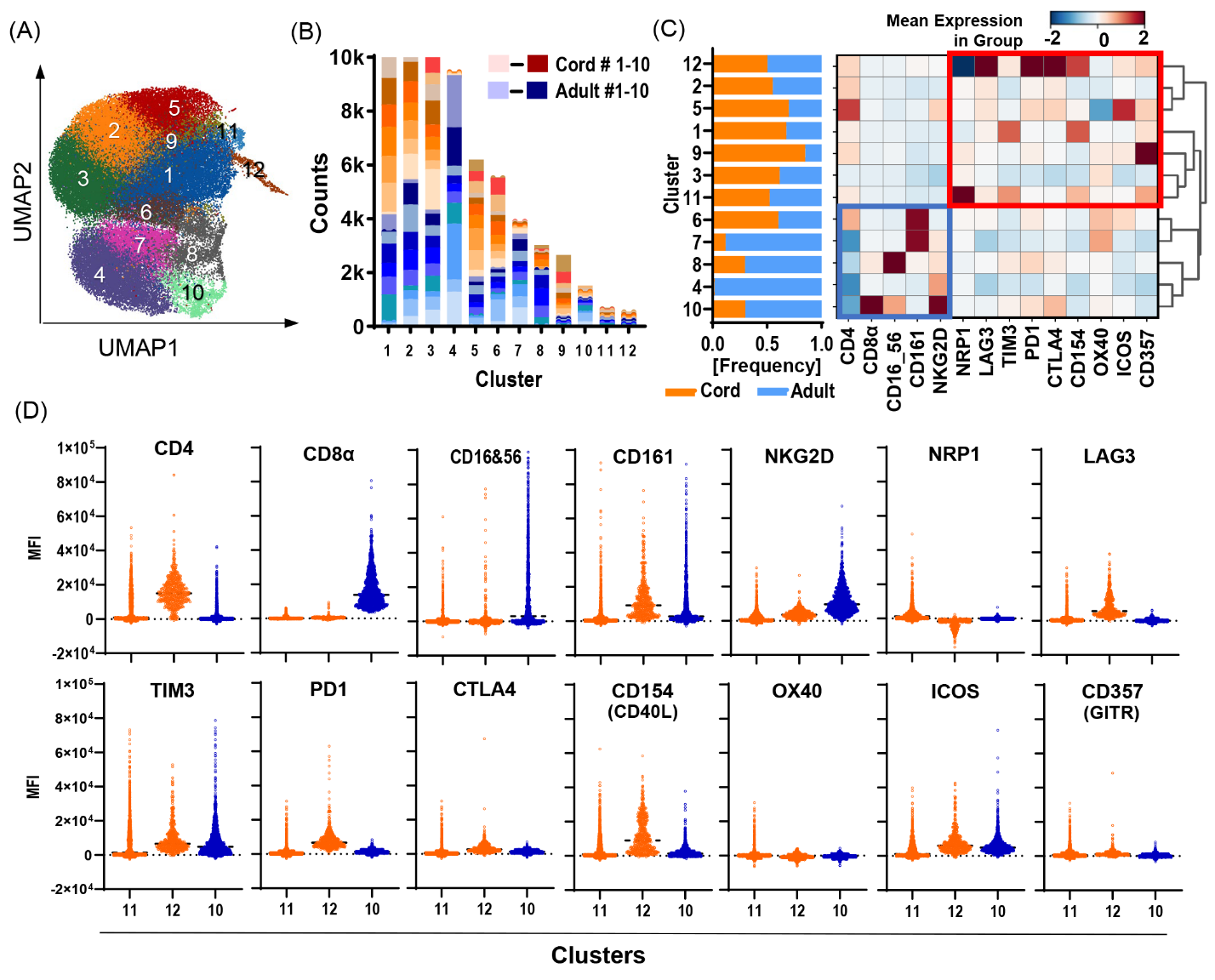


Supplementary Figure 4. Validation of effector and regulator iNKT cells from adult and cord-derived iNKT cells. *Ex vivo* expanded human iNK T cells were evaluated for various surface markers representative of effector or regulator phenotypes via multi-paremeter flow cytometry. (A) UMAP representation of flow cytometry clusters from adult and cord iNKT cells (3,500 cells from each donor, n=10 for each donor type, and 70,000 total iNKT cells). A total 13 distinct functional clusters were determined by the PhenoGraph algorithm (K=400). (B) A bar plot showing the frequency of iNKT cells belonging to each cluster and donor. (C) The distribution of adult vs cord-derived iNK T cells per each cluster, and Heatmap showing the mean expression levels of markers in each cluster. (D) Violin plot demonstrating the expression of surface markers on selected cluster, 11 and 12 as regulator subset, and 10 as effector subset. Crusty analysis of high dimensional cytometric data showed functional heterogeneity of iNKT cells, but segregated clusters (10, 4, 8, 7, 6) enriched with effector markers (CD16/CD56, NKG2D, CD161) from clusters (11, 9, 1, 5, 12) that were enriched with regulator markers.
